# A retrospective study on the incidence of medication-related osteonecrosis of the jaws (MRONJ) associated with different preventive dental care modalities

**DOI:** 10.1007/s00520-021-06587-x

**Published:** 2021-09-27

**Authors:** Christian Bacci, Alessia Cerrato, Elisa Bardhi, Anna Chiara Frigo, Selma Ahcene Djaballah, Stefano Sivolella

**Affiliations:** 1 Section of Clinical Dentistry, Department of Neurosciences, University of Padova, Via Giustiniani, 1, 35128 Padua, Italy; 2 Department of Cardiac, Thoracic, Vascular Sciences and Public Health, University of Padova, Padua, Italy; 3 UOC Oncology 3, Veneto Institute of Oncology IOV, IRCCS, Padua, Italy

**Keywords:** MRONJ, BRONJ Bisphosphonates, Cancer, Prevention, Oral health

## Abstract

**Purpose:**

To assess the efficacy of different preventive dental visits and treatments in reducing the risk of medication-related osteonecrosis of the jaws (MRONJ).

**Methods:**

In this retrospective study, patients diagnosed with MRONJ were divided into 5 groups based on available data: no preventive dental visits (group 0); dental visits and compliance with recommended treatments, at the university hospital’s dental clinic (group 1) or maxillofacial surgery unit (group 2), or at a private dentist’s (group 3); dental visits at one of the above and noncompliance with proposed treatments (group 4); patients judged eligible by the oncologist on panoramic radiography (group 5). Patients were classified on severity of MRONJ according to the Italian SIPMO/SICMF 2.0 staging system. A descriptive analysis was performed on the results. Fisher’s exact test was applied (*p* < 0.05).

**Results:**

Ninety-three patients diagnosed with MRONJ were considered for the study, but 22 were excluded due to a lack of data, leaving a sample of 71 cases. MRONJ staging was only 0 for some patients (26.92%) in group 0. In all groups, the majority of patients had stage 2 MRONJ. The proportions of cases in stage 3 were 7.69% in group 0, 18.18% in group 3, and 43.48% in group 5. Groups 0 and 3 were somewhat similar as regard MRONJ staging. Most patients in group 5 had MRONJ stage 2 or 3. No statistically significant differences emerged between the groups.

**Conclusions:**

Preventive dental care can reduce the risk of MRONJ providing patients comply with the specialist’s recommendations.

## Introduction

Medication-related osteonecrosis of the jaw bones (MRONJ) is one of the most debated diseases in dentistry. This adverse event was initially described as bisphosphonate-related osteonecrosis of the jaws (BRONJ) [1], but since 2010, there have been reports of this condition in patients taking other categories of drugs [2], making it necessary to rename the disease as medication-related osteonecrosis of the jaws (MRONJ), or simply ONJ [3]. The types of medication most frequently associated with ONJ contain two pharmacological agents [4]: antiresorptive drugs, including oral or intravenous bisphosphonates (BPs); and receptor activator of nuclear factor kappa-B ligand (RANK-L) inhibitors such as denosumab and antiangiogenics [5, 6]. The latter are monoclonal antibodies that block the receptor or growth factor (bevacizumab), and small molecules that take effect by binding the tyrosine kinase receptor (sunitinib and sorafenib) [7].

The sequence of events leading to the onset of MRONJ and the prevalence of the condition are still not completely clear [8]. Two classification systems are used in Italy nowadays to stage the disease: the AAOMS [3]; and the SICMF-SIPMO [4, 9]. Both classifications stage MRONJ on the basis of clinical and radiological criteria. Table 1 shows a comparison between the two classifications.

**Table 1:** Medication-related osteonecrosis of the jaws staging systems compared

Stage	AAOMS 2009 ^3^	SICMF-SIPMO 2020 ^9^
At risk category	No evidence of exposed or necrotic bone in patients who have been treated with bisphosphonates	
Stage 0	Nonspecific clinical findings and symptoms such as jaw pain or osteosclerosis, but no clinical evidence of exposed bone	
Stage 1	Exposed/necrotic bone in patients who are asymptomatic, with evidence of infection	Focal ONJ Clinical signs and symptoms: bone exposure, sudden dental mobility, non-healing post-extraction socket, mucosal fistula, swelling, abscess formation, trismus, gross mandibular deformity, and/or hypoesthesia/paresthesia of the lips CT findings: increased bone density limited to the alveolar bone region (trabecular thickening and/or focal osteosclerosis), with or without signs of: markedly thickened and sclerotic lamina dura, persisting alveolar socket, and/or cortical disruption 1A Asymptomatic 1B Symptomatic (pain and purulent discharge)
Stage 2	Exposed/necrotic bone associated with infection as evidence of pain and erythema of the region of exposed bone, with or without purulent discharge	Diffuse ONJ Clinical signs and symptoms: same as for Stage 1 CT findings: increased bone density extending to the basal bone (diffuse osteosclerosis), with or without signs of: prominence of the inferior nerve canal, periosteal reaction, sinusitis, formation of sequestra, and/or oroantral fistula 2A Asymptomatic 2B Symptomatic (pain and purulent discharge)
Stage 3	Exposed/necrotic bone in patients with pain, infection, and one or more of the following: pathological fracture, extraoral fistula, or osteolysis extending to the inferior border or sinus floor	Complicated ONJ Clinical signs and symptoms: same as for Stage 2, with one or more of the following: extraoral fistula, displaced mandibular stumps, nasal leakage of fluids CT findings: osteoscleorosis of adjacent bones (zygoma and hard palate), pathological mandibular fracture, and/or osteolysis extending to the sinus floor

MRONJ treatment strategies may be conservative, non-surgical, or surgical [10]. Non-surgical treatment is essentially aimed at controlling symptoms, generally pending spontaneous sequestration. It involves maintaining adequate oral hygiene, regular follow-up visits, and antibiotic and antiseptic therapy. Conservative treatment is sometimes combined with ozone therapy, hyperbaric oxygen therapy, and laser biostimulation [3, 4, 11]. Surgical treatment consists in surface osteoplasty, curettage, sequestrectomy, or bone resection. Surgery was initially reserved only for advanced cases [6, 12, 13], but some authors have claimed more recently that it can be effective in the early stages of the disease as well [14–16].

Risk factors for MRONJ are both local (dental infections, tooth extractions, oral surgery, ill-fitting dentures) and systemic (number of administrations of medication; duration of medical treatments; and, in the case of BPs, type of drug, as the risk of MRONJ is higher for zoledronic acid vs. pamidronate vs. other BPs) [17].

The main target of primary prevention in patients who are to be administered drugs potentially associated with the onset of MRONJ is to achieve and maintain a state of oral and dento-periodontal health and perfect oral hygiene, thereby eliminating local risk factors [18]. It has been demonstrated that preventive dental screening and treatment reduces the incidence of MRONJ [17, 19, 20]. Appropriate preventive oral surgery and conservative endodontic and prosthetic measures have been published [21–25].

A MRONJ risk reduction pathway has also been developed, based on a multidisciplinary collaboration between oncologists, maxillofacial surgeons, and dentists [26]. A standardized approach has yet to become well established, however. Differences in the professional profiles of the specialists performing the dental screening of patients for the prevention of MRONJ might influence their outcomes, in terms of both the risk of its onset and the staging of its severity. The purpose of this retrospective study is to seek any correlation between the modality of at-risk patients’ preoperative dental assessment and treatment planning and their MRONJ event and its staging.

## Materials and methods

Medical records of patients who developed MRONJ between 2010 and 2019 at Padua University Hospital and the Veneto Oncological Institute were retrospectively examined. The data were obtained from the e-Health Galileo computer system (NoemaLife, Bologna, Italy). For each patient, the following data were collected: age; sex; underlying diseases; comorbidities; types, route of administration, and duration of drug therapies; if and how a dental check-up was performed before starting drug therapies; and any local risk factors (e.g., periodontal and peri-implant disease or ongoing inflammation) [12, 18]. Data were also collected regarding the site of osteonecrosis, symptoms, SICMF-SIPMO staging, the treatment administered, and the outcome of this treatment.

The inclusion criteria were as follows: a previous intake of drugs associated with a risk of MRONJ and a diagnosis of MRONJ according to SICMF-SIPMO [9] recommendations; and availability of the data of interest. The exclusion criteria were as follows: previous head and neck radiation therapy, and unavailability of the data of interest.

Patients were divided into 6 groups, by modality of dental screening and treatment: Group 0 included patients who did not have a dental visit prior to being treated with the drug associated with a risk of MRONJ; Group 1 included patients preventively examined by dentists at the regional reference center for oral bone diseases (Padua University’s Dental Clinic), who were staged strictly according to the SICMF-SIPMO recommendations, and who adhered to the recommended treatments; Group 2 included patients who were preventively examined by specialists at Padua University’s Maxillofacial Surgery Units, and who adhered to the recommended treatments; Group 3 included patients who had a preventive dental check-up performed by their own dentists, and who adhered to the recommended treatments; Group 4 included patients who had a preventive dental visit with one of the previously mentioned specialists, but did not comply with the proposed treatments; and group 5 included patients who did not have a preventive dental visit, but who had been judged eligible for antiresorptive therapy by the treating oncologist based on a panoramic radiograph and a radiologist’s report.

The study protocol complied with the Declaration of Helsinki and was approved by the Ethics Committee for Clinical Trials (CESC) at Padua University Hospital (n°8628 10.02.2021).

### Statistical analysis

Since this is a retrospective study, a descriptive statistical analysis was performed on the data collected, using SAS 9.4 (SAS Institute Inc., Cary, NC, USA). Fisher’s exact test was used (*p* < 0.05).

## Results

Ninety-three patients diagnosed with MRONJ were considered for the study, but 22 were excluded due to a lack of data, leaving a sample of 71 eligible patients: 50 females (70.42%) and 21 males (29.58%), with a mean age of 68.7 years (SD 7 years). Patients had received a mean 20.53 cycles of therapy with zoledronate (4 mg once a month). Twenty-five patients (35.21%) switched from zoledronate to denosumab, receiving a mean 13.6 cycles of therapy (120 mg once a month). The reasons for the switching were bone progression or toxicity to zoledronic acid (e.g., renal failure). Sixteen patients (22.53%) were also treated with bevacizumab iv. (mean 7.5 mg/kg every 2/3 weeks) (Table 2).

**Table 2:** Mean, minimum, maximum, median, and quartile ratings for the study population’s age, number of treatment cycles, and duration of therapies

No. of patients	Variable	Mean	Minimum	Maximum	Median	Lower quartile	Upper quartile
71	Age (years)	68.7	44.0	90.0	70.5	61.5	78.0
71	Duration of therapy (months)	33.9	1.0	300.0	18.0	11.0	31.0
65	Bisphosphonates (cycles)	34.2	2.0	360.0	19.0	8.0	28.0
6	Denosumab (cycles)	14.7	1.0	34.0	12.0	7.0	25.0
25	Bisphosphonates switched to	21.5 BP	3.0 BP	67.0 BP	21.0 BP	6.5 BP	26 BP
	Denosumab (cycles)	13.6 D	1.0 D	34.0 D	11.0 D	6.0 D	17.5 D

The site of MRONJ was the maxilla in 24 patients (33.80%), the mandible in 44 (61.97%), and both jaws in 3 (4.23%). Patients were classified as SIPMO SICMF MRONJ Stage 1 in 25.35% of cases, Stage 2 in 54.93%, and Stage 3 in 19.72% of cases (Table 3).

**Table 3:** Patients’ MRONJ staging according to the SICMF-SIPMO criteria

Stage	No. of patients	%
1	18	25.35
2	39	54.93
3	14	19.72

The 71 patients in our study population were grouped as shown in Table 4, and the staging of MRONJ in each group of patients is shown in Table 5. As for their treatment, 51 patients (71.83%) were given antibiotics, 46 (64.78%) took painkillers, and 62 (87.32%) only used antiseptic therapy. Resection of osteonecrotic tissue was performed in 27 cases (38.02%), while spontaneous sequestration of the osteonecrotic area occurred in 9 (12.67%). Biopsy of the necrotic bone was performed in 24 cases, and aggregates of actinomycetes were identified at histology in 13 cases (54.17%) [27]. Following conservative or surgical treatments, 40 patients (56.33%) went into remission of their MRONJ, 28 (39.43%) relapsed, and the disease developed at a new site in 3 (4.22%). It was impossible to collect MRONJ treatment outcome data for 11 patients who died of their underlying disease.

**Table 4:** Patient distribution by preventive dental screening modality

Preventive dental screening modality	No. of patients	%
Group 0	26	36.62
Group 1	2	2.82
Group 2	4	5.63
Group 3	11	15.49
Group 4	4	5.63
Group 5	24	33.80

**Table 5:** Correlation between MRONJ staging and dental screening modality groups, with and Fisher’s exact test result

Prevention by stage				
Prevention				
Groups	Stage 1 (*n* cases, %)	Stage 2 (*n* cases, %)	Stage 3 (*n* cases, %)	Total
0	7 (26.92)	17 (65.38)	2 (7.69)	26
1	0 (0.00)	2 (100.00)	0 (0.00)	2
2	1 (25.00)	3 (75.00)	0 (0.00)	4
3	3 (27.27)	6 (54.55)	2 (18.18)	11
4	2 (50.00)	2 (50.00)	0 (0.00)	4
5	4 (17.39)	9 (39.13)	10 (43.48)	23
Total	17	39	14	71

## Discussion

The diagnosis of MRONJ is based on patients’ medical history, previous drug intake, and clinical and radiographic signs [28, 29]. MRONJ is a relatively recently identified disease, and this explains why the numerous studies conducted have reported very different prevalence rates, natural histories, and treatment proposals. Regarding the prevalence of MRONJ, it is also necessary to distinguish between cancer patients, for whom the risk ranges from 0.2 to 6.7%, and non-cancer patients, whose prevalence is relatively low (0–0.4%) ^3^.

The reported average age of patients with MRONJ is around 70 years old [30] (and it was 68.7 [SD = 7 years] in our sample). The disease occurs twice as often in the mandible as in the maxilla [31] (and in our sample, it affected the mandible in 44 cases, the maxilla in 24, and both jaws in 3 patients). There are discordant data regarding the prevalence of MRONJ by sex, but it seems to affect females more than males [32]. Here again, our sample reflects those published in the literature, with females (70.42%) more affected than males (29.58%).

In all the proposed protocols for managing MRONJ, prevention is the key factor. It has always been the most effective approach, not only to reduce the risk of the disease’s onset, but also to facilitate its early diagnosis, make the disease less disabling, and improve patients’ quality of life [14].

The aim of primary prevention is to eliminate local risk factors, i.e., all oral and dental disease, to restore and maintain a good state of oral health, and to prevent the onset of adverse events [14]. One of the first studies dealing with MRONJ prevention, published in 2009, demonstrated a 33% reduction in the incidence of MRONJ with appropriate preventive dental treatments [22]. Other authors reported a 50% reduction in the risk of developing MRONJ in patients who had a preventive dental check-up compared to those who did not [20]. Findings in the present study are comparable for the patients who did not have a check-up with an oral cavity specialist (i.e., 50 patients in group 0, and 5 vs. 21 belonging to groups 1–2–3–4) [33]. A meaningful reduction in the risk of MRONJ among multiple myeloma patients treated with BPs was confirmed if preventive measures had been adopted [17]. In our sample, MRONJ was observed in 3/21 patients (14.2%) who started BP treatment at a time before the risk of MRONJ had come to light; 2/20 patients (10%) who started taking BPs without any preventive dental check-up; and none of the patients whose BP therapy started only after preventive dental care measures (0%) [17].

The risk of developing MRONJ varies, depending on the type of drug prescribed, the cumulative dose, and the duration of the treatment, as well as concomitant risk factors, and the patient’s oral health. Physicians prescribing drugs associated with MRONJ should inform patients and their dentists about the type of drug to be administered, the dosage, the frequency of administration, and the related risk of adverse events. The dentist should then be able to identify the teeth with an uncertain prognosis before a patient’s drug therapy starts. Once their therapy has begun, dentists need to distinguish between which dental treatments are indicated (associated with a low risk of developing MRONJ, and useful for its prevention), possible (associated with a low risk of developing MRONJ, but unable to prevent it), or contraindicated (associated with a risk of developing MRONJ, and not essential).

According to the literature [34], zoledronic acid is the drug most involved in the onset of MRONJ. In our sample, zoledronic acid was the culprit in 80.28% of cases (57 patients), followed by alendronate in 4.22% of cases (3 patients), ibandronate and neridronate in 4.22% of cases each (3 patients each), and pamidronate in 1.40% (1 patient). These data are in line with previous studies [35, 36]. The patients in our sample had received a mean 20.53 injections of zoledronic acid (4 mg, once a month), corresponding to a typical treatment lasting for almost 2 years. The risk of developing MRONJ as a result of taking antiresorptive (e.g., denosumab) or antiangiogenic (e.g., bevacizumab) medication appears to be lower than for iv. BPs because the former have a shorter half-life (e.g., 20 days for bevacizumab) and do not accumulate in the bone.

The aims of the present study were to assess the efficacy of preventive dental care, but also to establish whether clinicians conducting preventive dental visits can play a role in reducing or increasing the risk of MRONJ. Various figures were involved, namely the dental team at Padua University’s Dental Clinic (a regional reference center for oral bone diseases) and Maxillofacial Surgery unit, patients’ own dentists, and the oncologist prescribing the drugs associated with the risk of MRONJ (who relied on dental X-rays). Patients who did not have a preventive dental visit and those who did, but failed to comply with the specialist’s recommendations, were also examined.

Most of the patients who developed MRONJ had not had a dental check-up before starting therapy with drugs associated with the risk of MRONJ (36.62%), confirming the association between the onset of MRONJ and the lack of a preventive dental visit. Much the same percentage of patients (33.80%) developed MRONJ after having their dental health checked not by a dentist or maxillofacial surgeon, but by a medical oncologist, who relied on a radiographic image and a radiologist’s report. This comes as no surprise, given the well-known importance in the onset of MRONJ of conditions that X-rays are unable to reveal, such as tropism of the mucous membranes, the patient’s oral hygiene, or the fit of removable prostheses [8]. Patients who had not had any preventive dental visit and those assessed by their treating oncologist presented with more severe osteonecrosis (more cases of MRONJ in stages 2 and 3 according to the SICMF-SIPMO criteria).

A very small number of patients with MRONJ (2.82%) had been previously examined at the Dental Clinic where the SICMF-SIPMO’s “Clinical—Therapeutic Recommendations on Osteonecrosis of the Jaw Bones (MRONJ) associated with drugs and its prevention” [9] were strictly followed. A slightly larger number of patients (5.63%) had been previously examined at the Maxillofacial Surgery Unit. The frequency of patients with MRONJ was 3–7 times higher in the case of preventive visits conducted by a general dentist (15.49%).

None of the patients previously seen by a dental specialist (hospital dentist, maxillofacial surgeon, or private dentist) developed MRONJ stage 3 (5 patients had stage 2 disease, and one had stage 1), while patients who had a preventive visit with their own dentist developed MRONJ stage 3 in 18.18% of cases (2 patients), stage 2 in 54.55% (6 patients), and stage 1 in 27.27% (3 patients) of cases.

In group 0 (no preventive dental visits), 7.69% of patients (2 patients) developed MRONJ stage 3, while in group 5 (patients assessed by their oncologist), 43.48% of patients (10 patients) developed MRONJ stage 3.

Based on the data emerging from this study, patients preventively examined by experienced dentists or maxillofacial surgeons have a much lower risk of developing MRONJ, and any cases that do occur are usually milder (in stages 1–2). It should be emphasized, however, that our results were not statistically significant.

Multiple healthcare professionals—dentists, physicians, oral oncologists, maxillofacial surgeons, and others involved in a patient’s care—can play a part in the prevention of MRONJ [25]. An ideal MRONJ risk reduction pathway should aim to enable a patient’s prompt access to high-quality preventive dental treatment and facilitate their access to local services for patients given some drug therapies for some cancers that expose them to a higher risk of developing MRONJ [26]. It should also ensure timely and effective communication between oncologists, maxillofacial surgeons, and dentists. It would be desirable for dentists in charge of oral health in patients who are candidates for the administration of drugs potentially associated with MRONJ to be part of a team with expertise on this disease. Such an ideal path comes up against an important obstacle in the need to proceed urgently with treatment in cancer patients. For instance, the data that emerged on analyzing our group 4 (patients not adhering to treatments proposed by a dental specialist) can be largely explained by the fact that the urgency of starting the cancer treatment drug is often incompatible with dental clinic waiting times. The same may apply to patients who do not have a preventive dental visit, or whose dental health is judged by the oncologist on the strength of a panoramic radiograph, or by a general dentist ill-prepared on the matter of MRONJ prevention.

This retrospective study has significant limitations regarding the unavailability of some data and the small sample size. Our findings nonetheless suggest not only that preventive dental care is necessary, but also that it should be managed by an experienced clinician and conducted according to the latest guidelines.

## Conclusions

Specialist preventive dental care has an impact in reducing the risk of MRONJ. The active collaboration of health professionals involved in the management of patients at risk or suffering from by MRONJ can help to minimize the disease’s occurrence and its sequelae.

## Data Availability

Data are available.
